# A Collection of Symptoms Going from Left to Right and from Right to Left

**Published:** 1891-03

**Authors:** John Dike

**Affiliations:** Melrose, Mass.


### A COLLECTION OF SYMPTOMS GOING FROM LEFT TO RIGHT AND FROM RIGHT TO LEFT.

John Dike, M. D., Melrose, Mass.

We so constantly hear of Lachesis being given for symptoms going from left to right and Lycopodium for those going from right to left, that we are apt to think that these two remedies are the only ones having this lateral peculiarity. A careful study of the materia medica, however, will reveal that there are many remedies that have the lateral characteristic more or less prominent. Even Lachesis, given as it always is, for symptoms of every kind going from left to right, nevertheless has one form of headache which goes from right to left.

In this paper the author has collected all the symptoms he could find, by a perusal of Hering's *Guiding Symptoms*, having the lateral character. It is hoped that the practical physician may find them useful at the bed-side.

### LEFT TO RIGHT.

### HEAD.

Headache: first on left side then on right; beginning in teeth and jaw, extending to ear, temple, and vertex: Zinc.
Headache in temples: Agar.
Headache: pain as if a knife were drawn through the head from l. to r.: Arn.
Headache, frontal: Carbol-ac.
Headache, frontal, with oppression of chest: Carbol-ac.
Headache, frontal eminence: Lycop-virg.
Headache l. to r.: Cimex, Nux-mos.
Headache, neuralgic: Cinchon.
Headache, lancinating: Elaps.
Headache, shooting pain: Eupat-perf.
Burning in head: Calc-ars.
Choreic jerking of head: Ign.
Drawing, painful, in forehead: Agar.
Erysipelas upon scalp, forming vesicles: Rhus-t.
Pressure in temples: Calad.

### LEFT TO RIGHT.

Tearing in cheeks, temples, and eyes, also in right side only : Verat.
Tearing in forehead : Lachnan.

### FACE.

Drawing pain in jaw : Oxal-ac.
Erysipelas of; redness of nose going from left side of nose to right : Hydrast.
Itching of; l. to r. : Pallad.
Neuralgic pains in : Cinchon.

### EYES.

Darting pains in : Croc.
Diseases going from l. to r. : Apis.
Fissures in external canthi : Nat-mur.
Pain around : Pallad.
Pain in : Mur-ac., Psor.
Pressure above eyes : Agar.
Shooting in eyes : Chel.
Weeping : feeling as if left eye had been weeping, going to right with corresponding appearances : Croc.

### EARS.

Aching in : Merc.
Earache : Calc-phos.
Earache ; sticking pain in l. ear, later in r. : Aloes.
Hemorrhage of : Merc.
Ringing and whistling in ears : Merc.
Shooting in : Merc.

### NOSE.

Coryza with running of water : Agar., Allium-c.
Discharge from nose : Lach.

### MOUTH.

Gums, swelling of : Nat-mur.
Teeth : pain in molars : Lycop-virg.

### THROAT.

Angina granulosa : Plumb.
Diphtheria developed : Lach., Petrol.

### LEFT TO RIGHT.

Pains from l. to r.: Lach., Lyc.
Tonsillitis: Plumb., Sabad., Spig.
Tonsillitis with yellow, granulous, follicular ulcers small and painful, with burning stinging pains: Plumb.
Ulceration in: Lach.

### CHEST.

Cardiac region; stitches in; going to r.: Arn.
Left chest to right shoulder; deep seated pains in: Eupat-perf.
Chest in general: Lil-t.
Clavicles, sore: Calc-phos.
Clavicles, stitches in extending to right side: Cornus.
Mammæ; abscess of: Arn.
Mammæ; scirrhus of: Brom.
Mammæ; stitches in nipples: Carduus-mar.
Pain in upper chest: Cimex.
Pain in, below heart: Gels.
Sore pain, deep in: Agar.
Sore spot in left side with lancinating pains going to r.: Calc-c.
Stitches in: Æsculus, Kreos.
Stitches in, during inspiration, Bry.

### ABDOMEN.

Aching: Nux-mos.
After pains in: Ipec.
Cutting pains in: Ipec.
Cutting pains in: Lachnan.
Drawing pain through pit of stomach: Card-mar.
Griping in: Agar.
Hard body seems to roll from navel, when turning from left to right: Lyc.
Lancinating pains in: Calc-c.
Ovarian region: fine cutting pain in left: when stretching in bed going across to right; first faint then stronger; increased after repeated stretching: Apis.
Ovarian tumors: left ovary first, later the right: Lach.
Pain in abdomen caused by a fall: Coloc.

### LEFT TO RIGHT.

Paroxysmal pains in : Coloc.
Pubic region : pain in, from l. to r., followed by earache : Calc-p.
Pubic region : scrotum : burning in : Lachnan.
Pubic region : testicles diminish in size first l. then r : Lyssin.
Renal region ; sharp shooting pains in : Kali-bichrom.
Rending pain across when lying in bed : Lyssin.
Tearing in hypochondriacal region : Lyc.

### BACK.

Neck : nape of : pressing pain in spot size of a coin going over to right side of neck : Carduus-mar.
Renal region : burning : Lachnan.
Renal region : shooting pains in, extending down to the thighs : worse on motion : Kali-bichrom.

### UPPER EXTREMITIES.

Clavicle : soreness of : Calc-phos.
Shoulder : deltoid muscle, twitching in : Oxal-ac.
Shoulder : neuralgic pain in : Lac-can.
Shoulder : pain in : Medorrhin.
Arms : drawing in upper : Calc-ars.
Arms : dull pain in : Calc-phos.
Arm ; dull pain in, worse from change of weather ; Calc-phos.
Arms : pain in : Formica-r.
Elbows : shooting through : Calc-phos.
Wrists : pains in : Kreos.
Hands : burning and itching in : Medorrhin.
Hands : electrical current : sensation of in : first l. then r. : Lil-tig.
Hands : itching of : Ars-iod.
Fingers : sensation of electric current in : first l. then r. : Lil-t.
Fingers : stitching in : Pallad.

### LOWER EXTREMITIES.

Hips in general : Arg-met.
Hip joints : tearing pain in : Ambra.
Hip bone : stitches in : Pallad.

### LEFT TO RIGHT.

Groin: left then right: cutting, drawing, aching, and soreness: Calc-phos.
Leg: left; hip, knee, and toes: pain passes to right thigh and ankle (gout): Benz-ac.
Legs: coldness of: Cup-s.
Legs: formication and pain in: Ars-iod.
Legs: œdematous: Lach.
Leg: rheumatic pains: Benz-ac.
Lower leg: sensation of splinter in: Agar.
Lower leg: pain in tibia: Agar.
Lower leg and feet; heat of: Cup-s.
Knees, pains in, when walking: Calc-phos.
Knees in general: Arg-met.
Feet; falling asleep of: Coloc., Millefol.
Foot: dry, scaly herpes on left internal malleolus and then upon right: Cactus-g.
Toe: great; pain in first joint of left great toe suddenly moving to corresponding joint of right toe: Eupat-perfol.
Toe: great; gout in: Dulc.
Toe: great; moving pain in: Eupat-perf.

### IN GENERAL.

Paralysis (poisoning): Acon.
Eruptions: Asimin-tr.
Seems to see a bright flame terminating in a point: Cinch-bol.
Pain in gout: Colch.
Objects appear to move: Lac-deflor.
Erysipelas spreads: Lach.
Rheumatic pain: Lach.
Pains in general: Lycop-virg.

### SYMPTOMS GOING FROM RIGHT TO LEFT.

### HEAD.

Aching; dull in frontal protuberance: Acet-ac.
Aching; headache r. to l.: Inula, Lachesis, Lyssin.
Headaches r., in A. M., l. in P. M.: Bov.

### RIGHT TO LEFT.

Head ; headache from vertex to left ear, then over head to right ear : Nit-ac.
Headache ; changing back and forth : Colch.
Headache ; acute boring pains through forehead, r. to l. : Iris-v.
Head ; crazy feeling in head : Lil-tig.
Head ; cutting in right occipital to left parietal bone : Bell.
Head ; drawing through side of head and neck to clavicle : Ind-met.
Head, is drawn to right side, later to left : Angust.
Pain in occiput : Psor.
Pain in right, then left temple : Fluor-ac., Ipomœa, Pallad.
Pressing pain ; Eupat-purp.
Pressing pain in frontal region, r. then l. : Colch.
Pressure in right orbital region, afterward on left also : Cainca.
Shocks : sudden, constrictive, in temples ; Plat.
Shooting in right temple, passing to left, Lil-tig.
Shooting like an electric shock from temple to occiput, Iris-v.
Stitches in forehead, Calc-ars.
Stitches in temples, Agar.

### FACE.

R. to L. : Cheek and left eye, Amyl-nit.
Erysipelas of face, Apis.
Erysipelas beginning in right ear and spreading over face, Sulph.
Neuralgia : supra-orbital, Natr-mur.
Pains in lower jaw, severe, Mez.
Pain over whole side of right face and suddenly springing to left side, Coccin-sep.
Prosopalgia, Mez.
Redness, dark-brown, in face, Anthrac.
Rhus poisoning in face, Crot-tig.
Swelling of parotid, Lac-c.
Swelling, œdematous, under eyebrows, Kali-c.

### EYES.

Ciliary blepharitis, Psor.
Erysipelas in right eye and then in left, Apis.

### RIGHT TO LEFT.

Inflammation of the eyelids, Bad., Psor.
Itching on border of eyelids, Alumen.
Pains in eyes, Chel.
Stitch, sudden, in front part of both eyes, fr. R. to L. Eyes running water, Chim-umb.
Ulcers on cornea, Con.
Dark veil passes before the eyes, Natr-mur.
Warm water, feeling of, flowing over eyes, Nit-ac.

### EARS.

Darting pain in ear, Dolich.
Earache, Angust.
Earlobe, affections of, going fr. R. to L., Arg-met.
Pinching in ears, Bell.
Stitches in ear, Arg-nit.

### NOSE.

Epistaxis, going from R. to L., Coca.
Pain from R. to L. over bridge of nose, Euphrasia.
Stoppage of nostril, Benz-ac.

### MOUTH.

Soreness of under-lip, Ars.
Stabbing pains in gums, Glon.
Swelling of gums, Ars.
Swelling of gums and soreness of under-lip, Ars-met.
Toothache, Acon.

### THROAT.

Diphtheritic patches spread, Lyc.
Eustachian tubes, Arg-met.
Pain in throat, Dolich.
Dull piercing pains in side of throat, Millef.
Rawness of palate, Lyc-virg.
Sore throat, Arum-tri., Baryt-c., Lyc., Podophyl., Sulph.
Swelling and inflammation of tonsils, Gels., Lach., Lyc.
Swelling of trachea, Arum-tr.

### CHEST.

Cramps in chest ; wakens at two A. M. : Lachnan.

### RIGHT TO LEFT.

Darting pain in hypochondria. Profuse sweat; had to bend double, clench hands, and writhe in agony: Calc-c.
Pain in thorax: Chel.
Pain from one axilla to the other: Elaps.
Painful spot on second rib to sternum: Bry.
Pleuritic pains: Bad.
Pneumonia: Chel.
Sensation as if something smooth were gliding from right hypochondrium to left: Daph-od.
Sharp pain with soreness to touch: Elaps-cor.
Stitches in lungs: Alumina.
Stitches in right hypochondria then in left: Brom., Thuja.

### ABDOMEN.

Burning or boring stitches in ovaries: Lyc.
Cutting belly-ache, right to left iliac fossa, thence to rectum: Sanguin.
Cutting across hypogastrium: Lyc.
Cutting stitches in lower abdomen: Merc.
Cutting pains across abdomen: Lyc.
Cutting jerks: Calc-ars.
Darting pain in testes: Lyc-virg.
Pains from right iliac region to left side of abdomen: Lac-c.
Pain above crest of ilium: Iris-v.
Pains, from r. to l.: Nicc.
Spasmodic pains in hypochondrium: Nux-mos.
Spasms in pit of stomach: Con.
Stitches across abdomen: Dros.
Sharp pains in lower abdomen: Cocc-cact.
Twitching in ovarian regions: Abrot.

### BACK.

Burning soreness in back from R. to L., Agar.
Cramp-like pains in neck from side to side, Calc-ph.
Erysipelas across back, Apis.
Pain in back, Lobel-cœr.
Stitches in scapula, Cocculus.

### RIGHT TO LEFT.

### UPPER EXTREMITIES.

Neuralgia of shoulder, Eup-purp.
Pain in shoulders, Apis.
Rheumatic pains in right shoulder going to left upper arm and elbow joint, Lobel.
Rheumatism in shoulders, Amm-mur., Apis, Lyssin.
Pains in paroxysms, first in right shoulder and arm, down side to hip then across to left hip, Lyc.
Numbness of hands, Cocc.
Hand: Pain in right hand goes to left arm and down elbow, thence to heart, later in right thigh and ankle, Benz-ac.
Pain from right hand to left arm, Benz-ac.
Hand cold, first right then left, Medorr.
Hand and arm stiff and painful, Lil-tig.
Dull aching in fingers, Abrot.
Fingers; panaritium going from r. to l., Sanguin.

### LOWER EXTREMITIES.

Hip; pain in, Lil-tig., Lyc.
Hip bone; right, then left; tearing in; extending to knee, Canth.
Hip; soreness and drawing pain in joint, Lil-tig.
Testicles, r. to l., dragging pain: Hydrast.
Shifting pains in leg, Hydrast.
Smarting of leg, Lil-tig.
Varicose veins inside of right thigh, then of left, Ferr.
Neuralgia of knee, Benz-ac., Eup-purp.
Pains in knees, Badiaga, Benz-ac.
Shooting from knees to ankles, Lyc.
Right, then left; tendo Achilles, pain in, Benz-ac.
Foot goes to sleep, Millef.

### IN GENERAL.

Right to left in general, Aspar, Carbol-ac., Rheum.
Neuralgia, now right, now left, Magn-phos.
Sensation as if something pressed downward on right side of body and coming up on left, Chim-mac.

### RIGHT TO LEFT.

Rheumatism and gout. Benz-ac.
Acute rheumatism : Chel.
Stinging, burning rheumatic pains with great soreness and lameness; profuse sweat relieves : Apis.
Stinging, itching on small places, Aur-mur.
Varicose veins, right, then left, Ferr.
Things whirl : Cocc.

